# 

**Published:** 1883-01

**Authors:** 


### CONTENTS.

### COMMUNICATIONS.

On Relations which Dental Caries—as discovered amongst the Ancient Inhabitants, &c.—may be supposed to hold to their Food and Social Condition ........ 1
A New Departure ........ 11
Flasks and Flasking ........ 16
Pyemia of Many Years' Standing from Concealed Abscess, caused by a Wisdom Tooth ........ 19

### SELECTIONS.

The Bacillus Tuberculosis and the Refutation of its Etiological Relation with Tuberculosis ........ 29
Gems ........ 31
The Bacillus ........ 31
Creosote Impurities ........ 34
Lesions of the Teeth in Locomotor Ataxy ........ 36
Tetanus—Its Diagnosis and Treatment ........ 36
Wheat-meal Bread as a means of Diminishing Tubercular Disease 38
Soldering Without an Iron ........ 40
Luxation of the Jaw ........ 41
Carbolic Acid ........ 42
Artificial Ivory ........ 42

### PROCEEDINGS.

The American Academy of Dental Science ........ 42
Mad River Dental Society ........ 43

### EDITORIAL.

Neuralgia—a Case ........ 45
A Mechanical Forceps ........ 47
Translation from a Paper ........ 48
Operative Dentistry ........ 48
Bibliographical ........ 49
Anæsthetics ........ 49
A New Editor ........ 50
Died ........ 50
Married ........ 50

